# Clinical phenotype, MRI inflammatory involvement, and treatment patterns in HLA-B27 positive and negative axial spondyloarthritis: a Saudi MRI-based cohort study

**DOI:** 10.1186/s41927-026-00632-0

**Published:** 2026-03-18

**Authors:** Abdallah Alqethami, Mohammed Basalama, Samiyah Alsaedi, Khulud Alqahtani, Eman Alsindi, Sabri Alsaeedi, Roaa Mahroos, Maysoon Taher

**Affiliations:** Rheumatology Department, King Fahad General Hospital, Jeddah, Saudi Arabia

**Keywords:** Axial spondyloarthritis, HLA-B27, MRI, diagnostic delay, CRP, Saudi Arabia

## Abstract

**Background:**

HLA-B27 is strongly associated with axial spondyloarthritis (axSpA) and influences disease phenotype, though its prevalence and clinical relevance vary by population. Magnetic resonance imaging (MRI)-based cohorts offer substantial information about early axSpA detection and phenotype expression. Data from Saudi Arabia remain limited.

**Objective:**

To compare clinical characteristics, diagnostic delay, inflammatory markers, MRI findings, and treatment patterns between HLA-B27-positive and HLA-B27-negative patients with MRI-confirmed axial spondyloarthritis.

**Methods:**

This retrospective cross-sectional study included adults with clinician-diagnosed axSpA fulfilling ASAS imaging criteria. Clinical features, C-reactive protein (CRP) levels, MRI findings, and treatment utilization were compared between HLA-B27 groups. Diagnostic delay was analyzed as continuous and categorical data.

**Results:**

Eighty-four patients were included (32 HLA-B27 positive; 52 HLA-B27 negative). HLA-B27 positive patients were more frequently male (*p* = 0.004) and had higher rates of uveitis (*p* = 0.006), family history of SpA (*p* < 0.001), and elevated CRP levels (*p* < 0.001). Diagnostic delay was prolonged in both groups. Median diagnostic delay did not differ significantly between groups (*p* = 0.48). Spinal inflammatory involvement on MRI was more frequent in HLA-B27 positive patients but did not reach statistical significance (*p* = 0.11). No significant differences in biologic treatment utilization were observed.

**Conclusion:**

In this MRI-confirmed axial spondyloarthritis cohort, HLA-B27 positive patients exhibited a more defined axial and extra-musculoskeletal phenotype with higher systemic inflammation. Peripheral manifestations, diagnostic delay, and biologic treatment patterns were broadly comparable between HLA-B27 subgroups. These findings highlight regional phenotype variation and support the continued role of MRI-based evaluation in axSpA.

## Introduction

Axial spondyloarthritis (axSpA), a form of spondyloarthritis (SpA), is a chronic inflammatory disease affecting the sacroiliac joints and spine, often associated with extra-musculoskeletal manifestations such as uveitis and enthesitis [1, 2]. The HLA-B27 allele plays a central role in disease pathogenesis and phenotype expression, although its prevalence and clinical significance vary considerably across populations [3, 4]. HLA-B27 prevalence is elevated in Northern European populations, whereas it is significantly lower in Middle Eastern countries, including Saudi Arabia, thereby diminishing its diagnostic efficacy [2, 3].

Large international cohorts, including DESIR, REGISPONSER, and large Chinese axSpA cohorts, have demonstrated that HLA-B27 positive axSpA is typically characterized by male predominance, younger age at onset, increased uveitis, and greater axial involvement [5–7]. Conversely, HLA-B27 negative axSpA presents with more clinical heterogeneity and diagnostic challenges due to its less typical disease patterns [2, 6].

Existing Saudi data remain limited. Omair et al. reported low HLA-B27 prevalence among Saudi axSpA [3], while Bedaiwi et al. identified wide variability in clinical presentation and emphasized CRP as a key marker of disease activity independent of HLA-B27 status [8]. However, no prior Saudi study has directly compared the HLA-B27 subgroup within an MRI-confirmed cohort nor examined MRI distribution patterns or diagnostic delay categories across HLA-B27 strata.

MRI plays an essential role in identifying early axSpA and detecting active inflammatory lesions before radiographic changes develop [4, 9]. MRI-based cohorts provide unique opportunities to study phenotype variation independent of structural progression, particularly in regions where HLA-B27 is less prevalent and diagnostic reliance on imaging is high [3, 4, 9]. Recent Gulf consensus recommendations further emphasize the central role of MRI as a first-line imaging modality in suspected axSpA within the Arabian Gulf region [10].

Therefore, this study aimed to compare clinical phenotype, diagnostic delay, CRP levels, MRI distribution and treatment patterns between HLA-B27 positive and HLA-B27 negative axSpA patients in a Saudi MRI-confirmed cohort. This work expands regional understanding of axSpA heterogeneity and addresses gaps identified in prior studies.

## Methods

### Study design and setting

This was a retrospective, cross-sectional study conducted at a tertiary rheumatology center in Saudi Arabia. The study aimed to compare clinical phenotype, diagnostic delay, inflammatory markers, MRI distribution, and treatment patterns between HLA-B27 positive and HLA-B27 negative patients with axial spondyloarthritis (axSpA).

### Patient selection and classification

Patients were identified through electronic medical records. All included patients had a clinical diagnosis of axSpA made by a consultant rheumatologist and fulfilled the Assessment of Spondyloarthritis International Society (ASAS) imaging arm criteria, defined by the presence of active sacroiliitis on magnetic resonance imaging (MRI).

Only patients meeting the imaging arm were included to ensure diagnostic specificity. Patients fulfilling only the ASAS clinical arm were not included, which may have preferentially excluded some HLA-B27 negative axSpA cases. Conventional pelvic radiographs were not consistently available; therefore, classification into radiographic (r-axSpA) and non-radiographic axSpA (nr-axSpA) according to modified New York (mNY) criteria could not be reliably performed and was not analyzed.

### Data collection

Demographic and clinical data were extracted from electronic records, including age at diagnosis, sex, inflammatory back pain, extra-musculoskeletal manifestations (uveitis, psoriasis, inflammatory bowel disease), peripheral manifestations (arthritis, enthesitis, dactylitis), and family history of spondyloarthritis.

### Diagnostic delay

Diagnostic delay was defined as the interval between the patient-reported onset of chronic back pain documented in the medical record and the date of a formal axSpA diagnosis by a rheumatologist. Delay was calculated in weeks and additionally categorized into clinically meaningful groups: < 1 year, 1–3 years, 3–5 years, and > 5 years. Categories were reported in years to aid clinical interpretation.

### Laboratory assessment

C-reactive protein (CRP) measured at the time of diagnosis was recorded as a marker of systemic inflammation.

### MRI acquisition and interpretation

MRI of sacroiliac joints was performed using a 1.5-Tesla scanner with standard protocol, including short tau inversion recovery (STIR) and T1-weighted sequences. Active sacroiliitis was defined according to ASAS/OMERACT criteria [9].

Spinal MRI was available for a subset of patients and was assessed for the presence of inflammatory lesions. Spinal inflammatory lesions were defined according to ASAS MRI criteria for inflammatory spinal lesions, including bone marrow edema consistent with active spondyloarthritis [4, 9]. Formal MRI scoring systems were not applied. MRI scans were interpreted as part of routine clinical care by experienced musculoskeletal radiologists; readers were not blinded to clinical information or HLA-B27 status.

### Treatment patterns

Biologic therapy was analyzed descriptively. Treatment exposure was defined as ever using a biologic agent during the disease course. The number of biologic agents used, and the frequency of individual agents were recorded. Treatment response and persistence were not evaluated due to the absence of standardized disease activity indices (e.g., ASDAS, BASDAI).

### Statistical analysis

Continuous variables were summarized using means (± standard deviation) or medians (interquartile range), as appropriate. Group comparisons were performed using the Mann-Whitney U test for continuous variables and the chi-square or Fisher’s exact test for categorical variables.

Multivariable regression analysis was not performed due to the limited sample size and the risk of model overfitting. The potential confounding effect of sex was therefore considered qualitatively and explicitly addressed in the discussion. A two-sided p-value < 0.05 was considered statistically significant.

### Ethical considerations

The study was approved by the Institutional Review Board of the hosting institution. As this study involved retrospective analysis of anonymized data, the requirement for informed consent was waived.

## Results

A total of 84 patients with MRI-confirmed axial spondyloarthritis were included in the analysis, comprising 32 HLA-B27 positive and 52 HLA-B27 negative individuals. Demographic, clinical, laboratory, imaging, and treatment characteristics were compared between groups.

### Demographic characteristics

Demographic characteristics are summarized in Table 1. The mean age at diagnosis did not differ significantly between HLA-B27 positive and HLA-B27 negative patients (30.68 vs. 31.83 years; *p* = 0.41). In contrast, sex distribution differed markedly: males accounted for 86.4% of the HLA-B27 positive group compared with 55.6% of the HLA-B27 negative group (*p* = 0.004).

**Table 1:** Demographic characteristics of axial SpA patients stratified by HLA-B27 status

Characteristics	HLA-B27 positive (*n* = 32)	HLA-B27 negative (*n* = 52)	*P*-value
**Age at diagnosis (years)**			0.41
Mean	30.6	31.8	
Median	29	30	
**Standard deviation**	6.8	6.3	
**Sex distribution**			0.004
Male (%)	86.3	55.5	
Female (%)	13.6	44.4	

### Clinical characteristics

Clinical findings are summarized in Table 2. Inflammatory back pain was reported in all patients. HLA-B27 positive patients demonstrated significantly higher frequencies of uveitis (47.7% vs. 16.7%; *p* = 0.006) and family history of spondyloarthritis (70.5% vs. 22.2%; *p* < 0.001). HLA-B27 positive patients also had a significantly higher mean number of SpA features (3.48 vs. 2.22; *p* < 0.001).

This difference in the total number of SpA features was mainly caused by axial and extra-axial components, such as uveitis and family history, rather than peripheral manifestations.

Consistent with this, peripheral arthritis, dactylitis, enthesitis, psoriasis, and inflammatory bowel disease (IBD) showed no significant differences between HLA-B27 groups.

**Table 2:** Clinical features of axial SpA patients according to HLA-B27 status

Clinical feature	HLA-B27 positive (*n* = 32)	HLA-B27 negative (*n* = 52)	*P*- value
**Diagnostic delay (weeks)**			0.48
Mean	288.4	341.9	
Median	208	260	
**Diagnostic delay categories (years)**			
< 1 year	15.6	13.5	
1–3 years	37.5	32.7	
3–5 years	15.6	23.1	
>5 years	31.3	30.8	
Family history of SpA (%)	70.4	22.2	< 0.001
Inflammatory back pain (%)	100	100	1.00
Arthritis (%)	47.7	33.3	0.18
Dactylitis (%)	18.1	11.1	0.38
Enthesitis (%)	36.3	33.3	0.79
Psoriasis (%)	9.1	5.5	0.57
Inflammatory bowel disease (%)	0	0	NA
Uveitis (%)	47.7	16.6	0.006
Number of SpA features *			< 0.001
Mean	3.4	2.2	
Median	3	2	

* Number of SpA features refers to the total count of ASAS-defined spondyloarthritis features present per patient at baseline

negative patients (*p* = 0.48). Delay categories showed similar distributions; more than 30% of both groups experienced delays exceeding five years

### Diagnostic delay

Diagnostic delay was prolonged in both groups. Median symptom duration before diagnosis was 208 weeks for HLA-B27 positive patients and 260 weeks for HLA-B27.

### Laboratory findings (CRP)

CRP levels were significantly higher among HLA-B27 positive patients (Table 3). Mean CRP was 17.95 mg/L compared with 6.90 mg/L in the HLA-B27 negative group (*p* < 0.001). Median CRP was also higher in the HLA-B27 positive group (12.35 vs. 5.02 mg/L).

### MRI findings

All patients had active sacroiliitis on MRI by inclusion criteria. Spinal inflammatory involvement on MRI was more frequent in HLA-B27 positive patients (50%) than in HLA-B27 negative patients (33.3%), although the difference did not reach statistical significance (*p* = 0.11) (Table 3).

**Table 3:** Laboratory and MRI findings according to HLA-B27 status

Feature	HLA-B27 positive (*n* = 32)	HLA-B27 negative (*n* = 52)	*P*- value
**CRP at diagnosis (mg/L)**			< 0.001
Mean	17.9	6.9	
Median	12.3	5.0	
Standard Deviation	17.9	7.2	
Minimum	1.1	1.5	
Maximum	85.0	46.4	
**MRI findings (%)**			- 0.11
Sacroiliac inflammation	100	100	
Spinal inflammation	50	33.3	

### Treatment patterns

Biologic therapy exposure was analyzed at the patient level (Table 4). Among the 84 patients receiving biologic treatment, 32 were HLA-B27 positive and 52 were HLA-B27 negative.

TNF inhibitors were the most frequently prescribed biologic category in both groups, administered to 96.9% of HLA-B27 positive patients and 90.4% of HLA-B27 negative patients. IL-17 inhibitors were more commonly used among HLA-B27 negative patients (25.0%) compared with HLA-B27 positive patients (12.5%). JAK inhibitors were prescribed in 9.4% of HLA-B27 positive patients and 15.4% of HLA-B27 negative patients. No statistically significant differences were observed in biologic category distribution between the two groups.

Adalimumab was the most frequently used individual biologic agent in both groups. The distribution of other individual agents is presented in Table 5.

**Table 4:** Biologic therapy distribution by HLA-B27 status

Category	HLA-B27 (+) (*n* = 32)	HLA-B27 (–) (*n* = 52)	*P*-value
TNF-inhibitors	31 (96.9%)	47 (90.4%)	0.49
IL-17 inhibitors	4 (12.5%)	13 (25.0%)	0.26
JAK inhibitors	3 (9.4%)	8 (15.4%)	0.52

**Table 5:** Individual biologic agents

Agent	HLA-B27 positive	HLA-B27 negative
Adalimumab	26 (81.2%)	41 (78.8%)
Infliximab	8 (25.0%)	4 (7.7%)
Etanercept	4 (12.5%)	8 (15.4%)
Certolizumab	2 (6.2%)	8 (15.4%)
Secukinumab	2 (6.2%)	7 (13.5%)
Ixekizumab	2 (6.2%)	9 (17.3%)
Tofacitinib	3 (9.4%)	7 (13.5%)
Upadacitinib	0	1 (1.9%)
Golimumab	0	1 (1.9%)

## Discussion

In this MRI-confirmed cohort of Saudi patients with axial spondyloarthritis (axSpA), HLA-B27 positivity was associated with a recognizable clinical phenotype characterized by male predominance, a higher frequency of uveitis, a stronger family history of spondyloarthritis, and higher CRP levels at diagnosis. These findings are consistent with genotype-phenotype associations reported in large international cohorts, including REGISPONSER, DESIR, and large Chinese axSpA cohorts, which have demonstrated that HLA-B27 positive axSpA is more frequently associated with axial and ocular involvement and increased inflammatory burden compared with HLA-B27 negative disease [5–7].

Given the cross-sectional design and modest sample size, the present findings should be interpreted as descriptive and exploratory, rather than confirmatory. Similar limitations have been acknowledged in other single-center and regional axSpA studies, particularly those focusing on populations with low HLA-B27 prevalence [1, 4, 11]. Accordingly, causal inferences regarding genotype-phenotype relationships or treatment-related outcomes cannot be made.

Peripheral manifestations, including peripheral arthritis, enthesitis, dactylitis, psoriasis, and inflammatory bowel disease, did not differ significantly between HLA-B27 positive and HLA-B27 negative patients. This observation aligns with prior evidence suggesting that HLA-B27 status has a stronger influence on axial and extra-musculoskeletal manifestations, particularly uveitis, than on peripheral disease features [5, 6].

CRP levels at diagnosis were significantly higher among HLA-B27 positive patients. Previous studies have similarly reported higher CRP concentrations in HLA-B27 positive axSpA, supporting the hypothesis that this genotype is associated with a more inflammatory disease phenotype [7, 8]. However, the marked sex imbalance between groups represents an important potential confounder, as male sex itself has been associated with higher CRP levels and greater axial involvement in axSpA [2, 5]. Due to sample size limitations, multivariable adjustment was not performed, and this potential confounding effect should be considered when interpreting the CRP findings.

There was a significant diagnostic delay in both HLA-B27 positive and HLA-B27 negative patients, with numerous individuals facing delays surpassing five years. This finding is consistent with international reports demonstrating persistent diagnostic delay in axSpA even in healthcare systems with access to advanced imaging [11–13]. The absence of a meaningful difference in diagnostic delay between HLA-B27 subgroups suggests that, in this setting, diagnostic pathways may be influenced more by system-level factors, such as referral patterns and MRI accessibility, than by genetic status alone. Regions with low HLA-B27 prevalence may have limited reliance on genetic testing, making this particularly relevant [1, 11, 14].

The exclusive inclusion of patients fulfilling the ASAS imaging arm represents both a methodological strength and an important limitation, as it restricts generalizability to the broader axial spondyloarthritis population. Restricting the cohort to MRI-confirmed sacroiliitis enhanced diagnostic specificity and reduced misclassification, which is especially valuable in early axSpA [4, 9]. However, this approach inherently excludes patients fulfilling only the ASAS clinical arm, particularly among HLA-B27 negative individuals, thereby introducing selection bias and limiting generalizability to the broader axSpA spectrum [4]. Consequently, the full heterogeneity of HLA-B27 negative axSpA may be under-represented in this cohort.

In addition, the lack of systematic pelvic radiographs precluded classification into radiographic and non-radiographic axSpA according to the modified New York (mNY) criteria, limiting comparison with cohorts stratified by structural damage [15]. MRI assessment of spinal inflammation was descriptive and did not employ formal scoring systems or blinded readings, which may have contributed to measurement variability and warrants cautious interpretation of observed trends.

Treatment patterns were analyzed descriptively and did not differ substantially between HLA-B27 subgroups. This is consistent with current clinical practice, where biologic therapy selection is primarily guided by disease burden and treatment availability rather than HLA-B27 status [6, 16]. In the absence of standardized disease activity indices and longitudinal follow-up, treatment exposure should not be interpreted as a surrogate for treatment response or disease severity.

The limited representation of Janus kinase (JAK) inhibitors in this cohort likely reflects restricted local availability during the study period and may have influenced the observed treatment distribution.

Despite these limitations, this study provides important region-specific insights. To our knowledge, it represents one of the few MRI-confirmed axSpA cohorts from Saudi Arabia, examining HLA-B27 related differences in clinical phenotype, inflammatory burden, diagnostic delay, and imaging distribution. When considered alongside data from other low HLA-B27 prevalence regions, including Latin America, the results suggest a broader understanding of how genetic and regional factors interact to shape axSpA presentation [17, 18].

In summary, HLA-B27 positive axSpA in this cohort was associated with a more classical axial and extra-musculoskeletal phenotype and higher systemic inflammation, while peripheral manifestations and treatment exposure were comparable across genotypes. Diagnostic delay remained substantial irrespective of HLA-B27 status. These findings underscore the importance of MRI-based diagnostic strategies and highlight the need for improved clinical pathways to facilitate earlier recognition of axSpA, particularly in regions with low HLA-B27 prevalence.

## Conclusion

In this MRI-confirmed axial spondyloarthritis cohort, HLA-B27 positive patients demonstrated a more defined axial and extra-musculoskeletal phenotype, including higher systemic inflammation. However, peripheral manifestations, diagnostic delay, and biologic treatment patterns were broadly comparable between HLA-B27 subgroups. These findings highlight regional phenotype variation and support the continued role of MRI-based strategies in the evaluation of axial spondyloarthritis.

## Data Availability

The datasets used and/or analysed during the current study are not publicly available due to institutional and ethical restrictions related to patient confidentiality but are available from the corresponding author on reasonable request.

## Declarations

### Use of artificial intelligence tools

No artificial intelligence tools were used in data analysis. AI-assisted language editing tools were used for language refinement only.
